# TUFM: a central regulator in mitochondrial quality control and beyond

**DOI:** 10.1038/s41420-026-03075-1

**Published:** 2026-03-28

**Authors:** Xuanyi Li, Liangjie Dong, Tian Xiao, Jiayi Chen, Hang Ye, Siyi Zhu, Fulin Chen, Yuan Yu

**Affiliations:** 1 Key Laboratory of Resource Biology and Biotechnology in Western China, Ministry of Education, School of Medicine, Northwest University, Xi’an, China; 2 Laboratory of Tissue Engineering, College of Life Sciences, Northwest University, Xi’an, China; 3 Provincial Key Laboratory of Biotechnology of Shaanxi, Northwest University, Xi’an, China

**Keywords:** Energy metabolism, Mitophagy, Immune evasion, Tumour biomarkers, Apoptosis

## Abstract

Tu translation elongation factor, mitochondrial (TUFM) is a highly conserved, nuclear-encoded GTPase that is indispensable for mitochondrial protein synthesis. Beyond this canonical function, TUFM has emerged as a central regulator of mitochondrial quality control (MQC), orchestrating mitochondrial biogenesis, dynamics, and mitophagy through a location-dictates-function paradigm. Its subcellular localization and activity are precisely regulated by post-translational modifications, including phosphorylation, lactylation, ubiquitination, and acetylation, which collectively dictate its functional outputs in cellular homeostasis and stress responses. TUFM also serves as a critical interface in host-pathogen interactions, where viruses often hijack its pro-mitophagic function to evade mitochondrial antiviral signaling. Functioning as a cellular fate switch, the TUFM-MQC axis determines context-dependent pathological outcomes: its hyperactivation promotes cell growth and fuels oncogenesis, whereas its deficiency exacerbates cell death and contributes to neurodegeneration, inflammatory damage, and metabolic dysfunction. This review synthesizes current mechanistic insights into TUFM as a central MQC coordinator and delineates how its functional imbalance redirects cellular trajectories toward survival or death. Deciphering the regulatory logic and spatiotemporal dynamics of this pivotal hub offers promising avenues for developing targeted strategies to restore cellular homeostasis across a spectrum of diseases.

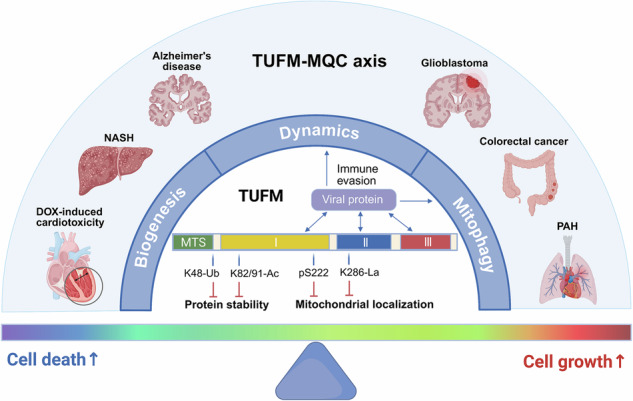

## Facts

- TUFM’s functional repertoire extends beyond mitochondrial translation to orchestrate mitochondrial quality control (MQC) processes, including mitochondrial biogenesis, dynamics, and mitophagy.
- A post-translational modification (PTM) network precisely tunes TUFM activity by modulating its subcellular localization and protein stability.
- Diverse viruses converge on hijacking TUFM to trigger mitophagy and dismantle the mitochondrial antiviral signaling platform.
- The TUFM-MQC axis exhibits context-dependent duality in determining cell fate: its hyperactivation promotes survival and oncogenesis, while its deficiency drives cell death and neurodegeneration.

## Open Questions

- What mechanisms govern TUFM’s cytosolic-mitochondrial shuttling, and how does its mislocalization drive pathology?
- How does TUFM sense cellular stress, prioritize, and coordinate distinct MQC processes to execute the final cell fate decision?
- What upstream regulatory networks decode TUFM expression, PTMs, and localization into disease-specific functional outputs?

## Introduction

Tu translation elongation factor, mitochondrial (TUFM) is a nuclear-encoded protein that is synthesized in the cytoplasm and subsequently imported into mitochondria. Studies of TUFM initially focused on *Saccharomyces cerevisiae* [1], and subsequently extended to *Arabidopsis thaliana* [2] and *Homo sapiens* [3]. As a core component of the mitochondrial translation machinery, TUFM is indispensable for mitochondrial protein synthesis [4]. However, recent research has expanded this canonical view, revealing TUFM to function as a central coordinating hub for mitochondrial quality control (MQC). Through dynamic subcellular localization, TUFM directly integrates and orchestrates key MQC processes, including mitochondrial biogenesis, dynamics, and mitophagy, thereby playing a decisive role in cellular metabolic adaptation and fate determination [5, 6].

While previous reviews have outlined the physiological and pathological functions of TUFM [7], a systematic synthesis of its role as an integrated coordinator remains lacking. Specifically, how its location-dictates-function logic orchestrates MQC, and how functional dysregulation explains its context-dependent roles across diseases, remain to be fully articulated. Therefore, this review aims to construct a unifying framework by systematically elucidating: (i) the molecular characteristics and post-translational modification (PTM) landscape of TUFM; (ii) its central role as an MQC integrator; (iii) its distinctive role in host-pathogen interactions; and (iv) how the dysregulation of the TUFM-MQC axis constitutes a core mechanism underlying cancers, neurodegenerative, metabolic, and cardiovascular diseases. Elucidating this framework is crucial for developing targeted therapeutic strategies aimed at modulating TUFM function.

## Characterization of TUFM

TUFM proteins are evolutionarily conserved, with their eukaryotic forms originating from the bacterial translation elongation factor Tu (EF-Tu). Compared to their bacterial homologs, the most distinctive features of eukaryotic TUFM are the acquisition of a mitochondrial targeting sequence (MTS) for mitochondrial localization and the loss of the KxKFxR motif present in bacterial EF-Tu, which can activate immune responses [8, 9]. One plausible evolutionary scenario suggests that bacterial EF-Tu was incorporated into host cells and subsequently adapted to become TUFM. This transition from “cytosolic immune activation” to “mitochondrial functional execution” is postulated to have facilitated viral hijacking of TUFM for immune evasion [10].

**Fig. 1:**
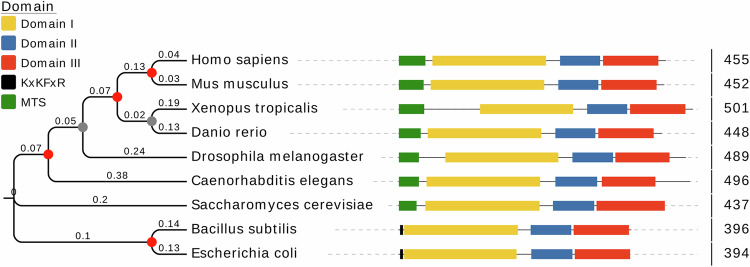
Phylogenetic analysis of TUFM homologs. A neighbor-joining phylogenetic tree of TUFM homologs was constructed in MEGA and visualized in Evolview [104]. Branch lengths scale with genetic distance, and nodal support is indicated by filled circles (red: bootstrap value = 100; gray: 90-99). Domain annotation using InterPro [105] identified Domain I (PF00009), II (PF03144), and III (PF03143). Notably, the KxKFxR motif is absent in all eukaryotic TUFM homologs. The mitochondrial targeting sequence (MTS) was predicted with TargetP-2.0 (https://services.healthtech.dtu.dk/services/TargetP-2.0/).

Structurally, TUFM comprises three characteristic domains (Fig. 1): the N-terminal GTP-binding domain (Domain I), the central aminoacyl-tRNA (aa-tRNA) binding domain (Domain II), and the C-terminal domain (Domain III) [11]. These domains collectively form the molecular basis for its function and interactions. Domain I is not only central to its canonical translation function but also serves as a key interface for interactions with autophagy-related proteins (e.g., the ATG12-ATG5 conjugate) and viral effectors such as influenza A virus (IAV) PB1-F2 protein [10, 12]. Domain II mediates important interactions, including its binding to human parainfluenza virus type 3 (HPIV3) M protein [13]. Although less extensively studied, Domain III has also been implicated in specific interactions, as evidenced by its binding to respiratory syncytial virus (RSV) NS1 protein [14]. This multi-domain, multi-interface plasticity provides TUFM with the capacity to sense and integrate diverse molecular signals, thereby establishing a structural foundation for its role as a coordinating hub in MQC.

## Post-translational modifications of TUFM

TUFM activity, which is essential for cellular homeostasis, is precisely regulated by PTMs that modulate its localization, stability, and interactions via multiple mechanisms (Table 1).

**Table 1:** Post-translational modifications of TUFM.

PTM type	Site	Species (Enzyme)	Functional Outcome	Molecular Mechanism	Core Aspect	References
Phosphorylation	Thr61, Thr382	*E. coli* (Doc)	Inhibits protein synthesis	Prevents ternary complex formation	Translational control	[15–17]
Phosphorylation	Thr63	*B. subtilis* (YabT)	Inhibits protein synthesis	Blocks EF-Tu release from the ribosome	Translational control	[18]
Phosphorylation	Tyr266	Bovine (c-Src)	Inhibits mitochondrial translation	Inhibits ternary complex formation	Translational control	[19]
Phosphorylation	Ser222	Human (PINK1)	Inhibits mitophagy	Retains TUFM in the cytosol	Subcellular localization	[5]
Lactylation	Lys286	Mouse (n.d.)	Inhibits mitophagy	Blocks mitochondrial import of TUFM	Subcellular localization	[6]
Ubiquitination	Lys27	Hamster (RNF185)	Promotes mitophagy	Recruits SQSTM1/p62 to engage LC3	Autophagic signal	[21]
Deubiquitination	Lys48	Human (USP5)	Stabilizes TUFM	Antagonizes proteasomal degradation	Protein stability	[22]
Deacetylation	Lys82, Lys91	Human (MRG15)	Promotes TUFM degradation	Enhances ClpXP-mediated degradation	Protein stability	[23]

*n.d.* not determined.

### Phosphorylation

Phosphorylation is an evolutionarily conserved regulatory mechanism for TUFM. In prokaryotes, phosphorylation of bacterial EF-Tu directly abolishes its translation function. For example, in *Escherichia coli*, phosphorylation at Thr61 and Thr382 prevents the formation of the ternary complex (EF-Tu•GTP•aa-tRNA), thereby inhibiting protein synthesis [15–17]. The Gram-positive bacterium *Bacillus subtilis* utilizes the kinase YabT to phosphorylate EF-Tu at Thr63 under nutrient starvation, blocking its release from the ribosome, which induces global translational arrest and metabolic dormancy [18]. In eukaryotic cells, this regulation of core translation function is maintained: c-Src-mediated phosphorylation at Tyr266 similarly impedes mitochondrial protein synthesis by inhibiting ternary complex formation [19]. By contrast, phosphorylation at Ser222 catalyzed by the kinase PINK1 represents a significant functional expansion—this modification does not directly affect translation but acts as a critical subcellular localization switch, promoting TUFM cytosolic retention and thereby inhibiting its pro-mitophagic activity [5]. This illustrates an evolutionary shift in the role of phosphorylation from a conserved translational switch to a versatile regulator of TUFM localization and function.

### Lactylation

Lactylation is an emerging PTM in which lactate-derived lactyl groups are covalently attached to lysine residues on target proteins, primarily catalyzed by certain acetyltransferases. This modification translates metabolic signals into functional changes in protein activity and gene expression [20]. Under pathological conditions such as traumatic brain injury (TBI), lactate accumulation drives TUFM lactylation at Lys286 [6]. This modification disrupts TUFM binding to translocase of outer mitochondrial membrane 40 (TOMM40), thereby impeding its mitochondrial import. Similar to PINK1 phosphorylation, the resulting cytosolic retention deprives TUFM of its mitophagic function, leading to accumulation of damaged mitochondria. Genetic evidence supports this mechanism: the non-lactylatable TUFM-K286R mutant maintains normal mitochondrial localization and autophagic capacity, whereas the wild-type protein loses function under these pathological conditions [6]. Therefore, Lys286 lactylation acts as a key molecular switch that converts metabolic disturbance signals (lactate accumulation) into MQC failure, ultimately leading to apoptosis. However, the specific enzymes responsible for this critical modification remain to be identified.

### Ubiquitination

Ubiquitination confers distinct functional fates upon TUFM through different chain linkages. For example, Lys27-linked polyubiquitination, catalyzed by the E3 ubiquitin ligase RNF185 through the interaction between its transmembrane domain and TUFM, functions as an “eat-me” signal for selective autophagy. This signal is recognized by the autophagy receptor sequestosome 1 (SQSTM1/p62), which recruits the autophagy machinery and promotes mitophagy [21]. Conversely, Lys48-linked polyubiquitination predominantly regulates TUFM stability, targeting cytosolic TUFM for proteasomal degradation. The ubiquitin-specific protease 5 (USP5) antagonizes this process by removing Lys48-linked ubiquitin chains, thereby stabilizing TUFM; in colorectal cancer, this stabilization is crucial for cell growth [22]. Thus, ubiquitination operates as a multifaceted regulatory system, capable of either actively initiating specific quality control programs via Lys27-linked chains or dynamically controlling TUFM abundance via Lys48-linked ubiquitination and its removal.

### Acetylation

Acetylation is another key mechanism regulating TUFM protein stability, particularly controlling its abundance within the mitochondrial matrix. The N-terminus of TUFM contains several conserved lysine residues, among which the acetylation status of Lys82 and Lys91 is a primary determinant of its stability. Upon deacetylation at these sites, TUFM is recognized by mortality factor 4-like protein 1 (MORF4L1/MRG15) and degraded by the mitochondrial matrix protease ClpXP [23]. Genetic evidence supports this regulatory logic: mutants mimicking constitutive deacetylation (K82R/K91R) undergo continuous degradation even in the absence of MRG15, whereas wild-type TUFM requires MRG15 for degradation and is stabilized upon MRG15 loss. This pathway directly couples the acetylation status within mitochondria to protein turnover. For example, in non-alcoholic steatohepatitis (NASH), upregulation of MRG15 promotes TUFM degradation, thereby triggering inflammatory responses [23]. Currently, both the precise enzymatic mechanism by which MRG15 promotes TUFM deacetylation and the structural basis for ClpXP recognition of the deacetylated form remain to be elucidated.

### The integrated regulatory network of TUFM PTMs

The expanding repertoire of PTMs targeting TUFM reflects its functional evolution from a core translation factor to a master coordinator of MQC. This sophisticated network converges on two principal regulatory dimensions: subcellular localization and protein stability. Localization is governed by specific PTMs that act as molecular switches. Ser222 phosphorylation and Lys286 lactylation enforce TUFM cytosolic retention, effectively uncoupling it from its mitochondrial functions. Stability, on the other hand, is regulated by compartmentalized degradation signals: Lys48-linked ubiquitination drives cytosolic turnover via the proteasome, while Lys82/91 acetylation status governs stability within the mitochondrial matrix, with deacetylation licensing ClpXP-mediated proteolysis. Beyond these axes, Lys27-linked ubiquitination directly tags TUFM as a substrate for selective autophagy, encoding a pro-mitophagic signal. Collectively, this multi-layered PTM network enables precise, context-dependent tuning of TUFM availability for MQC. Elucidating the spatiotemporal logic of this network, particularly the signals governing TUFM dynamics, is essential for understanding its context-dependent functions in health and disease.

## TUFM as the integrative hub of MQC

**Fig. 2:**
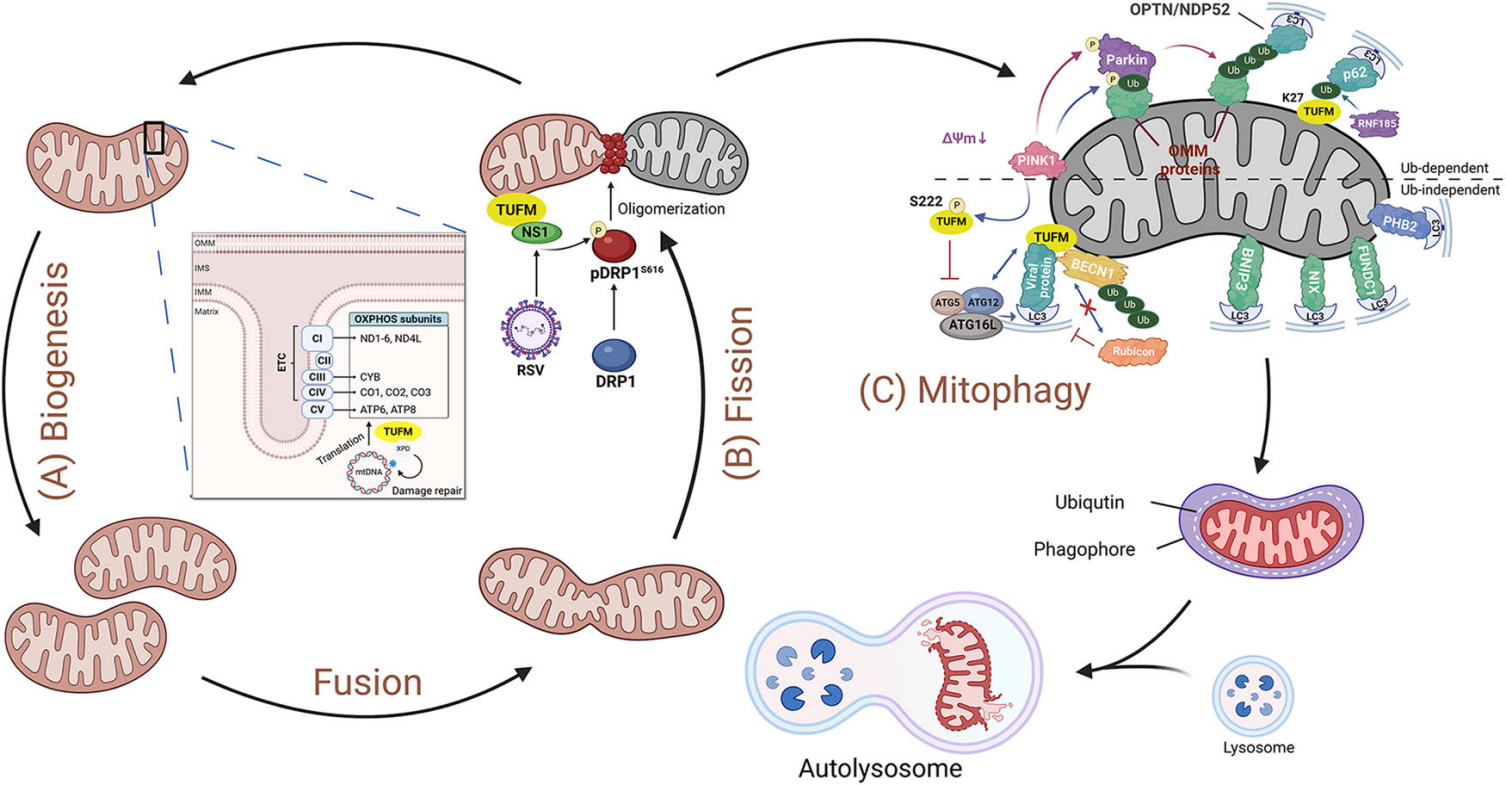
Role of TUFM in mitochondrial quality control. **A** TUFM coordinates mitochondrial biogenesis by facilitating the translation of mtDNA-encoded OXPHOS subunits and maintaining mtDNA integrity via DNA damage repair. **B** TUFM serves as a molecular bridge, transducing viral signals to DRP1-dependent mitochondrial fission. **C** TUFM orchestrates mitophagy through multiple mechanisms. While PINK1-mediated phosphorylation at Ser222 redistributes TUFM to the cytosol to inhibit mitophagy by blocking ATG12-ATG5 assembly, RNF185 ubiquitinates TUFM at Lys27 to promote SQSTM1/p62-LC3-mediated mitophagy. Alternatively, TUFM on the outer mitochondrial membrane can recruit core autophagy machinery to drive mitophagy in a ubiquitin-independent manner.

Mitochondria are essential for a variety of cellular functions, yet the accumulation of dysfunctional mitochondria can be detrimental to cells. This detriment arises primarily through excessive reactive oxygen species (ROS) production and mitochondrial DNA (mtDNA) release, which trigger oxidative stress, hyper-inflammatory responses, or dysregulated apoptosis [24–26]. To counteract these effects, MQC is maintained through a balance of mitochondrial biogenesis, dynamics, and mitophagy [27, 28]. These processes are coordinately regulated by TUFM to ensure cellular mitochondrial homeostasis (Fig. 2).

### Mitochondrial biogenesis

Mitochondrial biogenesis, defined as the process by which cells increase their mitochondrial mass and number, is essential for maintaining mitochondrial function and overall cellular health. This process involves mtDNA replication, mitochondrial protein synthesis, and their incorporation into the pre-existing mitochondrial network, given that mitochondria cannot be generated de novo [27, 28].

TUFM orchestrates mitochondrial biogenesis primarily via mitochondrial protein synthesis. As an evolutionarily conserved GTPase, it promotes the GTP-dependent delivery of aa-tRNA to the mitoribosomal A-site [11, 29, 30]. This facilitates mitochondrial mRNA decoding and translation elongation, generating essential oxidative phosphorylation (OXPHOS) subunits of Complex I (MT-ND1-6 and MT-ND4L), Complex III (MT-CYB), Complex IV (MT-CO1-3), and Complex V (MT-ATP6 and MT-ATP8) [31, 32]. Consequently, TUFM-mediated protein synthesis is indispensable for OXPHOS functionality and respiratory capacity [33].

Beyond mitochondrial protein translation, TUFM is pivotal for maintaining mtDNA integrity. While dispensable for mtDNA replication [34], it plays a key role in repairing oxidative DNA damage [35]. TUFM directly interacts with xeroderma pigmentosum group D (XPD) localized to mitochondria, a helicase that facilitates repair of mtDNA lesions via the nucleotide excision repair (NER) pathway, thereby preserving mitochondrial genome stability [35]. Thus, TUFM integrates dual biogenesis roles, facilitating OXPHOS subunit synthesis while ensuring mtDNA integrity.

### Mitochondrial dynamics

Mitochondrial dynamics, comprising fusion and fission, plays a critical role in maintaining MQC and cellular homeostasis [36]. Fusion facilitates the exchange of mitochondrial contents, allowing functional components to compensate for damaged ones within the organelle. Conversely, mitochondrial fission, primarily regulated by dynamin-related protein 1 (DRP1), segregates mitochondria into discrete units to enable selective removal of damaged organelles [37]. Proper regulation of these processes is essential to prevent the accumulation of damaged mitochondria, thereby maintaining energy production and meeting cellular metabolic demands [38].

Recent studies have identified TUFM as a key regulator of mitochondrial fission, particularly in the context of virus-host interactions. During RSV infection, TUFM functions as a mitochondrial receptor that recruits the viral NS1 protein to the outer mitochondrial membrane (OMM), enabling NS1-mediated DRP1 phosphorylation at Ser616 and subsequent mitochondrial fission [14]. Genetic evidence indicates that TUFM ablation prevents NS1 localization to mitochondria and consequently blocks DRP1 phosphorylation, demonstrating that mitochondrial recruitment of NS1 by TUFM is necessary for this viral modulation of mitochondrial dynamics [14].

### Mitophagy

Mitophagy, a selective form of autophagy, is a process that targets dysfunctional mitochondria for degradation, thereby maintaining a healthy mitochondrial pool essential for cellular homeostasis [39]. This quality control mechanism relies on molecular “eat me” signals displayed on damaged mitochondria, which are specifically recognized by autophagy machinery to ensure targeted sequestration into autophagosomes and subsequent lysosomal degradation. These recognition signals operate through two principal pathways: ubiquitin-mediated tagging and receptor-mediated surface labeling [40].

#### Ubiquitin-mediated mitophagy

The PINK1/Parkin pathway represents the best-characterized ubiquitin-mediated mitophagy mechanism [41]. Under normal conditions, PINK1 is constitutively imported into mitochondria and degraded [41, 42]. However, upon mitochondrial depolarization (ΔΨm loss), PINK1 accumulates on the outer membrane, activating its kinase function [43–45]. Active PINK1 phosphorylates ubiquitin and the E3 ligase Parkin, triggering Parkin’s mitochondrial recruitment and activation [46–48]. Parkin then ubiquitinates OMM proteins, providing a binding platform for essential autophagy adapters like optineurin (OPTN) and nuclear dot protein 52 kDa (NDP52), which initiate mitophagosome formation [49–51]. Paradoxically, although TUFM is a direct PINK1 substrate, its phosphorylation at Ser222 by PINK1 redistributes it to the cytosol, where it inhibits mitophagy by blocking ATG12-ATG5 complex assembly [5]. Conversely, in a PINK1-independent mechanism, the E3 ubiquitin ligase RNF185 ubiquitinates TUFM at Lys27. This modification facilitates TUFM’s interaction with SQSTM1/p62, which bridges the ubiquitinated TUFM to LC3 and thereby promotes mitophagy [21].

#### Ubiquitin-independent mitophagy

Mitophagy can also be triggered by ubiquitin-independent receptor proteins embedded in mitochondrial membranes. OMM receptors, including BCL2 interacting protein 3 (BNIP3) [52], BCL2 interacting protein 3 like (BNIP3L, also known as NIX) [53], and FUN14 domain containing 1 (FUNDC1) [54], directly bind to LC3 via a canonical LC3-interacting region (LIR) motif. This interaction engages the autophagy machinery and facilitates the recruitment of mitochondria into phagophores. Recent studies have also identified inner mitochondrial membrane (IMM) mitophagy receptors such as prohibitin 2 (PHB2) [55]. Upon mitochondrial depolarization and proteasome-dependent rupture of OMM, these IMM receptors bind to LC3 through the LIR motif to initiate mitophagy. Moreover, an expanding repertoire of mitophagy receptors has been revealed [56, 57]. TUFM emerges as a notable and paradoxical case, as it lacks a canonical LIR domain despite its close association with mitophagy.

The functional diversity of TUFM is closely associated with its subcellular localization. TUFM is primarily imported into the mitochondrial matrix, where it functions as a key component of the mitochondrial translation apparatus [5]. However, a minority of TUFM is localized on the OMM, making it more susceptible to proteinase K digestion compared to inner and matrix proteins [5, 58]. The OMM-localized TUFM operates as a mitophagy adapter by directly interacting with cytosolic autophagy machinery, including the ATG12-ATG5 conjugate, to drive the clearance of damaged mitochondria [5, 10, 58, 59]. Beyond this canonical role, TUFM recruits Beclin-1 to mitochondria, promoting its polyubiquitination to disrupt interactions with Rubicon, a well-established inhibitor of autophagy [60], thereby facilitating autophagosome biogenesis [61].

### TUFM as a dynamic MQC coordinator

TUFM operates through a location-dictates-function logic to coordinate MQC. Within the mitochondrial matrix, TUFM drives biogenesis via protein synthesis and mtDNA repair; upon translocation to the OMM, it acts as a mitophagy adapter or ubiquitination substrate to initiate organelle clearance. This dynamic localization, regulated by PTMs and protein interactions, enables TUFM to flexibly allocate MQC resources in response to cellular energy status and stress signals.

The balance of these processes determines cellular fate. Proper biogenesis coupled with timely damage clearance maintains cellular homeostasis and survival. Conversely, disruption of this equilibrium—such as aberrant cytosolic retention of TUFM leading to loss of its mitochondrial localization—results in defective autophagy and metabolic collapse, thereby steering the cell toward death. Thus, TUFM serves as a core signaling hub that integrates diverse upstream cues and directs specific MQC programs, ultimately governing the cell fate decision between survival and death.

## TUFM in host-pathogen interactions and viral immune evasion

**Fig. 3:**
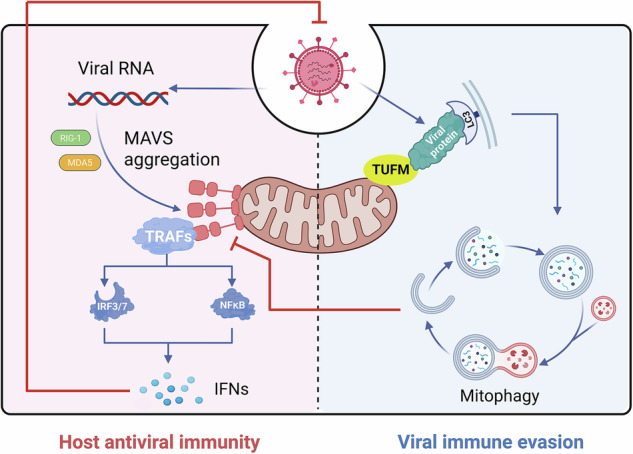
TUFM is hijacked as a pivotal node for viral immune evasion. **Left**, host antiviral immunity: Cytosolic sensors such as RIG-I and MDA5 detect viral RNA and trigger MAVS aggregation on the mitochondrial outer membrane. This nucleates a signaling platform that activates IRF3 and NF-κB via TRAF adapters, culminating in IFN production to establish an antiviral state. **Right**, viral immune evasion: Diverse viral proteins exploit TUFM to induce mitophagy. This leads to the degradation of the MAVS platform, effectively attenuating IFN responses and facilitating immune escape.

TUFM, a central executor of MQC, maintains cellular homeostasis by eliminating damaged mitochondria through mitophagy. Crucially, this homeostatic process is exploited by invading pathogens as a key mechanism of immune evasion. The strategic targeting of TUFM by viruses primarily orchestrates the dismantling of the mitochondrial antiviral signaling (MAVS) platform, a critical hub for initiating innate immunity. This platform assembles when cytosolic sensors, such as retinoic acid-inducible gene I (RIG-I) and melanoma differentiation-associated protein 5 (MDA5), detect viral RNA, triggering MAVS aggregation on the OMM. Aggregated MAVS recruits downstream adapters (e.g., TRAFs) to activate interferon regulatory factor 3/7 (IRF3/7) and NF-κB transcription factors, culminating in the production of type I interferons (IFNs) and the establishment of an antiviral state [62–65]. To evade this defense, numerous viruses exploit TUFM-mediated mitophagy for the “bulk clearance” of MAVS platforms (Fig. 3 and Table 2).

**Table 2:** Viral proteins targeting TUFM and recruiting autophagy machinery.

Virus (Protein)	TUFM Interaction Interface	Autophagy Recruitment Mechanisms	References
HTNV (Gn)	TUFM (n.d.) ↔ Gn (n.d.)	Gn LIR (YRTL) ↔ LC3B	[67]
IAV (PB1-F2)	TUFM (Domain I) ↔ PB1-F2 (C-terminal 50 residues)	PB1-F2 LIR (WLSL) ↔ LC3B	[12]
SFTSV (NP)	TUFM (N-terminal domain) ↔ NP (N-terminal, aa 2-92)	NP LIR (WSRI) ↔ LC3B	[68]
RSV (NS1)	TUFM (Domains I-III) ↔ NS1 (n.d.)	NS1 LIR (FVHV) ↔ LC3B	[14]
HPIV3 (M)	TUFM (Domain II) ↔ M (C-terminal 40 residues)	M (Lys295) ↔ LC3B	[13]
SVA (2 C)	TUFM (aa 56-252, E196/E211) ↔ 2 C (aa 231-321)	TUFM (Lys27-Ub) ↔ SQSTM1/p62 ↔ LC3B	[21]
HHV-8 (vIRF-1)	TUFM (n.d.) ↔ vIRF-1 (DRMs)	vIRF-1 (aa 227-236) ↔ GABARAPL1	[58, 69, 70]

*n.d.* not determined.

### Viral strategies for TUFM hijacking

A conserved strategy involves viral proteins directly binding TUFM to enable their own mitochondrial translocation and concomitantly recruiting LC3, typically through a canonical LIR motif (W/YxxL/I) [66], to initiate phagophore engulfment and MAVS degradation. This mechanism is employed by diverse viruses. For example, the glycoprotein (Gn) of Hantaan virus (HTNV) interacts with TUFM and LC3 via a C-terminal LIR motif (YRTL) [67]. In a similar manner, the PB1-F2 protein of IAV binds Domain I of TUFM through its C-terminal 50 amino acid residues while recruiting LC3 through its own LIR motif (WLSL) [12]. Likewise, the nucleoprotein (NP) of severe fever with thrombocytopenia syndrome virus (SFTSV) acts as a virulence factor by binding the N-terminal domain of TUFM through its own N-terminal region (aa 2-92) and concurrently recruiting LC3 via an N-terminal LIR motif (WSRI) [68]. In RSV, the NS1 protein binds TUFM (Domains I-III) to achieve mitochondrial localization and acts as an autophagy adapter, interacting with LC3 via its LIR motif (FVHV) to induce PINK1/Parkin-independent mitophagy [14].

Viruses can employ sophisticated mechanisms beyond direct LIR-mediated LC3 recruitment. HPIV3 similarly employs the M protein for TUFM binding and LC3 recruitment, albeit without a canonical LIR motif. Instead, residue Lys295 within the M protein C-terminus is critical for LC3 interaction and M-mediated PINK1/Parkin-independent mitophagy [13]. Seneca Valley virus A (SVA) utilizes its 2 C protein (aa 231-321) to bind the aa 56-252 region of TUFM (with key residues Glu196 and Glu211), recruiting the E3 ubiquitin ligase RNF185 to catalyze Lys27-linked polyubiquitination of TUFM. The ubiquitinated TUFM is then recognized by the selective autophagy receptor SQSTM1/p62, which bridges the complex to LC3, leading to autophagosomal degradation of MAVS [21].

Other viruses assemble multiprotein complexes incorporating TUFM and host autophagy factors to coordinate immune suppression. Human herpesvirus 8 (HHV-8) deploys viral interferon regulatory factor 1 (vIRF-1), which engages detergent-resistant membranes (DRMs) to recruit TUFM and NIX, forming a mitochondrial complex that promotes ATG12-ATG5 conjugation [58]. Within this complex, vIRF-1 recruits the ATG8 family member GABARAPL1 via its aa 227-236 region, while NIX stabilizes vIRF-1 aggregates to enhance mitophagic MAVS degradation [69, 70]. Similarly, vesicular stomatitis virus (VSV) exploits host NLR family member X1 (NLRX1); TUFM bridges NLRX1 to the ATG12-ATG5-ATG16L1 conjugate, enhancing autophagy while suppressing MAVS-mediated IFN-I responses [10].

### Host restriction and functional duality of TUFM

While TUFM predominantly facilitates viral immune evasion, it can paradoxically function as a host restriction factor in certain viral contexts. This is exemplified by avian influenza virus (AIV) carrying PB2 with the characteristic host-determining residue 627 [71]. Specifically, AIV with PB2-627E exhibits restricted replication in human cells, while human-adapted strains possess PB2-627K. Mechanistic studies reveal that TUFM exhibits high affinity for the avian signature PB2-627E protein. TUFM sequesters PB2-627E at mitochondria and facilitates its selective autophagic degradation. Consequently, TUFM knockout significantly enhances replication of PB2-627E viruses in human cells, whereas TUFM overexpression exerts a potent antiviral effect [71]. This selective restriction highlights TUFM’s novel role as a species barrier molecule against zoonotic influenza viruses.

TUFM’s functional duality in host-pathogen interactions extends beyond viruses to fungal infections. In *Histoplasma capsulatum*-infected macrophages, the TUFM-NLRX1 interaction promotes LC3-associated phagocytosis and proinflammatory cytokine production [72]. Conversely, TUFM expression contributes to the virulence of fungi like *Paracoccidioides brasiliensis*, potentially by aiding survival within macrophages [73]. These findings underscore the highly context-dependent nature of TUFM’s roles across diverse pathogen encounters.

### Convergent targeting and therapeutic implications

The recurrent targeting of TUFM by diverse viruses for MAVS degradation reflects a common exploitable vulnerability, rooted in TUFM’s unique molecular design (Table 2). First, its multi-domain architecture offers versatile docking platforms, allowing unrelated viruses to bind distinct interfaces (e.g., Domain I by IAV PB1-F2, Domain II by HPIV3 M). Second, its strategic OMM localization enables viral proteins to tether MAVS while recruiting autophagy machinery through their own LIR motifs. Third, its intrinsic pro-mitophagic properties allow direct co-option through TUFM-centric mechanisms, such as virus-induced ubiquitination or assembly into autophagy-promoting complexes.

Thus, TUFM serves as a shared vulnerability node in mitochondrial antiviral immunity due to its structural plasticity, exploitable function, and critical localization. This “multi-interface input, unified output” paradigm not only explains its broad viral targeting but also reveals a rational avenue for intervention. Disrupting virus-specific TUFM interfaces, such as those in Domain I or II binding pockets, may offer a strategy to block this immune-evasion pathway, though avoiding disruption of essential host functions remains a formidable challenge.

## Pathological roles of TUFM in human diseases

**Fig. 4:**
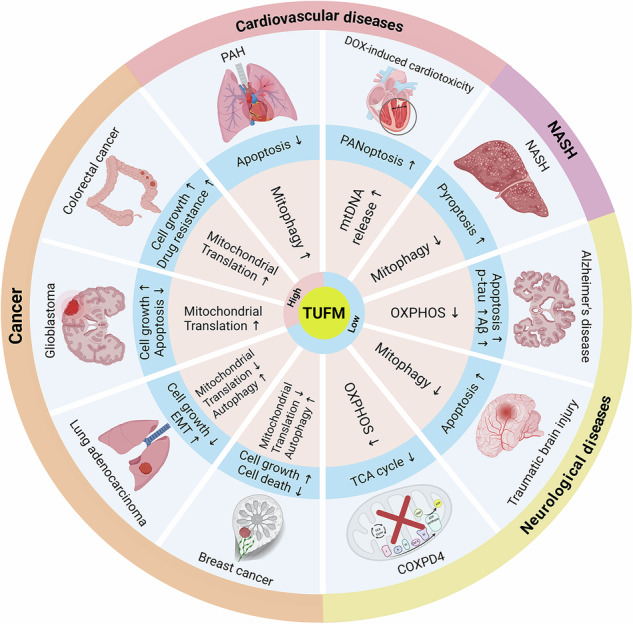
The TUFM-centered MQC network governs cell fate and disease outcomes. TUFM acts as a central node coordinating key MQC processes, including mitochondrial biogenesis, mitophagy, and OXPHOS. Altered TUFM expression perturbs this network, diverting cell fate toward pathological phenotypes such as uncontrolled proliferation, apoptotic death and inflammatory death (e.g., pyroptosis and PANoptosis), epithelial-to-mesenchymal transition, and metabolic dysfunction, which ultimately underlie the development of associated human diseases.

Dysregulation of the TUFM-MQC axis underlies multiple pathological conditions, spanning cardiovascular diseases, metabolic liver disease, cancer, and neurodegeneration (Fig. 4).

### Cardiovascular diseases

TUFM emerges as a critical regulator of MQC within the cardiovascular system, exerting significant influence on disease progression by modulating regulated cell death (RCD) pathways. Given that unresolved mitochondrial damage propagates detrimental RCD cascades, TUFM functions as a key sentinel by deploying mitophagy, regulating mitochondrial dynamics, and maintaining organellar integrity to constrain RCD activation and promote cellular survival.

Pulmonary arterial hypertension (PAH) is a severe cardiovascular disorder characterized by obstructive pulmonary vascular remodeling, increased pulmonary vascular resistance, and right ventricular failure [74]. In this condition, elevated TUFM expression in pulmonary arterial smooth muscle cells (PASMCs) enhances mitophagy, which suppresses apoptosis and thereby promotes excessive PASMC proliferation and vascular remodeling. Conversely, TUFM knockdown restores apoptosis sensitivity and normalizes mitophagic flux, thereby attenuating PAH development, a finding that highlights its therapeutic potential [75].

Beyond PAH, TUFM’s ability to suppress inflammatory RCD is crucial in protecting against chemotherapy-induced cardiotoxicity. The chemotherapeutic agent doxorubicin (DOX) induces dose-dependent cardiotoxicity, involving cardiomyocyte death driven by mitochondrial dysfunction. This death manifests as PANoptosis, a coordinated proinflammatory pathway integrating pyroptosis, apoptosis, and necroptosis [76, 77]. Mitochondrial damage releases mtDNA, which acts as a damage-associated molecular pattern (DAMP); this can activate inflammatory cascades leading to PANoptosome formation. Critically, TUFM counteracts DOX-induced cardiac injury by interacting with FUNDC1, thus preserving mitochondrial integrity, preventing mtDNA release, and blocking DAMP-driven activation of the PANoptotic cascade [78]. These findings underscore TUFM’s role as a master regulator suppressing inflammatory cell death in cardiomyocytes and highlight its significant therapeutic potential for mitigating DOX-associated cardiomyopathy.

### Non-alcoholic steatohepatitis

Transitioning from cardiovascular disorders to liver pathology, TUFM’s regulation of inflammatory RCD pathways also critically influences NASH pathogenesis, where hepatocyte pyroptosis (a lytic, inflammatory form of cell death [79]) contributes significantly to disease progression. This process is driven by the upregulation of MRG15 in both human and murine livers. Mitochondrial MRG15 interacts with and deacetylates TUFM, triggering its proteasomal degradation via the mitochondrial ClpXP protease complex [23]. TUFM degradation impairs mitophagy and activates the NLR family pyrin domain containing 3 (NLRP3) inflammasome [80], a key instigator of hepatocyte pyroptosis. This cascade fuels hepatic inflammation and fibrosis, thereby establishing a direct mechanistic link between MRG15-mediated TUFM loss and NASH pathogenesis. Consequently, therapeutic strategies targeting the MRG15-TUFM-NLRP3 axis hold promise for mitigating inflammatory liver injury in NASH and related metabolic liver diseases.

### Cancer

TUFM exhibits context-dependent oncogenic or tumor-suppressive functions across cancer types, determined by the metabolic dependencies and mitochondrial translation requirements of specific malignancies. In colorectal cancer, TUFM functions as an oncogenic driver. TUFM protein levels increase progressively from normal mucosa through adenoma to carcinoma, in parallel with increasing dysplasia severity [81]. Elevated TUFM expression is robustly associated with poor clinical outcomes, increased five-year recurrence rates, and serves as an independent prognostic factor [82]. This upregulation of TUFM protein represents a cytoprotective response, compensating for defective OXPHOS by enhancing mitochondrial protein translation efficiency [82, 83]. Mechanistically, TUFM upregulation, driven either post-transcriptionally by microRNA-451a downregulation or post-translationally through USP5-mediated deubiquitination, promotes colorectal cancer cell growth and confers resistance to chemotherapy [22, 84].

Similarly, elevated TUFM levels are observed in glioblastoma tumor tissues and glioblastoma stem cells (GSCs) compared to normal brain tissue [85]. Targeting TUFM, either with nanobodies or through genetic inhibition, suppresses GSC proliferation and induces apoptosis [85, 86]. This effect is primarily mediated by inhibiting TUFM-driven mitochondrial translation. Supporting this mechanism, the mitoribosome-targeting antibiotic combination quinupristin/dalfopristin, which blocks mitochondrial translation, similarly suppresses GSC growth [86]. Therapeutic strategies targeting mitochondrial translation, often involving TUFM downregulation or degradation, extend beyond glioblastoma. For example, the resveratrol analog HS-1793 downregulates TUFM and impairs mitochondrial function, leading to increased apoptotic death in breast cancer cells [87]. Similarly, ClpP activators ONC201 and TR-107 induce ClpP-mediated TUFM degradation, suppressing breast cancer cell proliferation and tumor growth [88, 89]. These approaches exploit the dependence of certain cancers on high TUFM levels and active mitochondrial translation.

Conversely, TUFM expression is reduced in other malignancies. In lung adenocarcinoma, TUFM protein levels decrease significantly with advancing tumor stage, correlating with disease progression [33]. Paradoxically, although TUFM knockdown reduces in vivo tumor growth, it promotes epithelial-to-mesenchymal transition (EMT). This pro-metastatic shift occurs via suppression of mitochondrial translation, concomitant with increased glycolysis, ROS production, and activation of the AMPK-GSK3β pathway [33]. This finding aligns with observations that TUFM is reduced during TGF-β1-induced EMT in hepatocytes [90]. Furthermore, TUFM loss in lung adenocarcinoma stimulates autophagy [33], a finding that contrasts with its canonical roles.

Mitochondrial translation defects linked to impaired TUFM function are also observed in specific breast cancer subtypes. In estrogen receptor-positive breast tumors, the frequent deficiency of core-binding factor subunit beta (CBFB) disrupts TUFM’s tethering to mitochondrial mRNAs, leading to defective mitochondrial translation and reduced ATP production. As a compensatory response, tumor cells exhibit enhanced AMPK activity, which in turn triggers cytoprotective autophagy [91]. Consequently, pharmacologic autophagy inhibition using hydroxychloroquine induces significantly greater cell death in CBFB-deficient compared with CBFB-proficient breast cancer cells [91], suggesting therapeutic potential for autophagy inhibition in cancers harboring mitochondrial translation defects.

Notably, TUFM expression varies markedly even within specific cancer types, as evidenced by comparisons across different cell lines. In hepatocellular carcinoma, TUFM expression is decreased in HepG2 cells but increased in HCC-S102 cells [92]. Similarly, gastric cancer cell lines show marked variation: AGS, SGC-7901, MKN-45, and KatoIII cells exhibit high TUFM expression, while MKN-28 and MKN-74 cells show low expression [92, 93].

In summary, TUFM exhibits a context-dependent dual role in cancer—promoting either cell survival or suppressing tumor growth—that underscores the profound importance of its precise regulation within the tumor microenvironment. Current evidence suggests that this opposing functionality is closely tied to its expression levels. Although the downstream mechanisms through which TUFM influences tumor fate—primarily via mitochondrial translation, metabolic reprogramming, and autophagy—are being delineated, the upstream regulatory networks that govern its heterogeneous expression remain largely elusive. Elucidation of these upstream regulatory switches is therefore essential for understanding how TUFM is directed toward oncogenic or tumor-suppressive programs and for identifying actionable therapeutic targets.

### Neurological diseases

TUFM is increasingly recognized as a significant factor in the pathophysiology of Alzheimer’s disease (AD) and cognitive aging [94, 95]. Notably, TUFM expression is markedly reduced in the brains of individuals with AD [96]. This reduction is also observed in aged APP/PS1 mice, a model of AD, where hippocampal TUFM mRNA and protein levels are decreased [94, 97]. This downregulation contributes to AD pathology through interconnected mechanisms. TUFM knockdown impairs mitochondrial respiratory chain activities, which in turn elevates ROS levels [33]. Elevated ROS enhances β-amyloid converting enzyme 1 (BACE1) RNA stability and translation, consequently elevating BACE1 protein levels and the catalytic product amyloid-β [94, 98]. Furthermore, TUFM knockdown promotes apoptosis and enhances tau phosphorylation. Importantly, the mitochondria-targeted antioxidant TEMPO mitigates these detrimental effects, underscoring the critical mediating role of ROS in these processes [94]. The therapeutic potential of targeting TUFM is further supported by the natural flavonoid kaempferide, which facilitates autophagic clearance of pathological tau via TUFM modulation [99].

Beyond its role in chronic neurodegenerative diseases, TUFM critically contributes to acute brain injury pathology. In TBI, pathological lactate accumulation drives extensive protein lactylation. Notably, TUFM is lactylated at Lys286, a modification that impairs its mitochondrial import [6]. Consequently, TUFM is retained in the cytoplasm, which abolishes its mitophagic function, leading to the accumulation of damaged mitochondria and subsequent neuronal apoptosis. Corroborating this mechanism, in vivo studies demonstrate that mice expressing the non-lactylatable TUFM-K286R mutant retain mitochondrial TUFM with functional mitophagy, thereby exhibiting significantly improved neurological recovery post-injury. Thus, Lys286 lactylation in TBI acts as a maladaptive switch, converting TUFM from a neuroprotective to a pro-apoptotic factor and highlighting it as a promising therapeutic target. Consistent with this, mild hypothermia mitigates neuronal damage by suppressing TUFM lactylation and restoring mitophagic flux [6].

Additionally, pathogenic *TUFM* variants cause combined oxidative phosphorylation deficiency 4 (COXPD4), a severe infantile encephalopathy characterized by early-onset brain malformations, diffuse white matter disease, and profound neurological impairment [4, 100, 101]. The underlying mitochondrial dysfunction in COXPD4 stems directly from TUFM deficiency, which impairs translation of mitochondrial-encoded OXPHOS proteins. This destabilizes respiratory complexes (particularly I and IV) and triggers degradation of nuclear-encoded subunits [102], leading to impaired OXPHOS function, TCA cycle inhibition, and elevated ROS levels [4, 103]. Zebrafish *tufm* mutants faithfully recapitulate key features of COXPD4, demonstrating analogous defects in mitochondrial respiration and thereby validating the critical role of TUFM in neuronal development and function [101, 103].

### Functional imbalance of the TUFM-MQC axis and pathological outcomes

Across various diseases, the pathological contribution of TUFM can be attributed to a core mechanism: functional imbalance of the MQC network coordinated by TUFM (Fig. 4). TUFM is not simply a “good” or “bad” protein—rather, it functions as a key regulatory hub that maintains dynamic equilibrium between mitochondrial function and cellular fate determination. The functional state of the MQC network is governed collectively by TUFM expression levels, PTM profiles, and subcellular localization. This state determines the direction and intensity of the network’s output.

In the context of different diseases, this balanced system exhibits a shift in direction, resulting in divergent pathological outcomes. In diseases such as colorectal cancer and glioblastoma, the TUFM-MQC axis shifts toward cell survival and growth. Aberrantly high expression or stabilization of TUFM promotes excessive mitochondrial biogenesis and anti-apoptotic mitophagy, thereby providing sustained metabolic support and resistance to cell death for tumor cells. Conversely, in neurodegenerative diseases, NASH, and certain cancers (e.g., a subset of lung adenocarcinomas), the axis shifts toward cellular decline and death. Loss of TUFM expression, functional inactivation (e.g., cytosolic retention due to lactylation), or abnormal degradation leads to impaired mitochondrial turnover, functional collapse, and accumulation of toxic byproducts (e.g., ROS, damaged mtDNA), thereby triggering inflammation, apoptosis, or pyroptosis.

Therefore, the onset and progression of these diseases can, in essence, be viewed as distinct collapses of the TUFM-MQC dynamic equilibrium system under specific pathological pressures. Restoration of this balance—that is, the precise enhancement or inhibition of TUFM’s function according to disease type—represents a common therapeutic rationale across diseases. Future therapeutic strategies will depend on accurately assessing the axis imbalance in specific diseases or even individual patients, and recalibrating TUFM activity accordingly. This perspective unifies the understanding of TUFM’s complex roles in disease and provides a theoretical framework for its translational potential as a tunable therapeutic target.

## Conclusion and perspectives

Originally defined by its role in mitochondrial translation, TUFM has emerged as a dynamic integrative hub that executes the core logic of the TUFM-MQC axis. Within this framework, diverse upstream signals (e.g., metabolic stress or pathogenic mutations) precisely regulate TUFM’s expression levels, PTM status, and subcellular localization, thereby functionally encoding its activity. Depending on its specific state, TUFM functions as a central dispatcher that preferentially directs specific MQC programs, including direct involvement in mitochondrial biogenesis, dynamics, and mitophagy. The ultimate output of the MQC network fundamentally determines cellular fate trajectories, steering cells toward homeostasis, proliferation, death, or malignant transformation. Consequently, functional dysregulation of TUFM, whether through hyperactivation or deficiency, constitutes a central mechanism linking diverse pathologies ranging from cancer to neurodegenerative diseases.

Despite significant progress in our understanding, translating the multifaceted biology of TUFM into clinical strategies faces several pivotal challenges. First, the regulatory logic and pathological consequences of its subcellular distribution remain incompletely understood. Recent findings suggest that TUFM can be retained in the cytoplasm, thereby challenging the prevailing paradigm of its exclusive mitochondrial localization [5]. The signals governing its cytosolic-mitochondrial shuttling, and whether cytosolic TUFM possesses independent signaling functions, represent crucial gaps in our understanding of its pathological roles. Second, it is essential to elucidate how TUFM integrates and prioritizes different MQC pathways to function as a final switch for cell fate. This necessitates a shift from examining individual branches to conducting systems-level investigations of how TUFM coordinates with other quality control processes, including mitochondrial fission and fusion. Finally, the upstream regulatory codes dictating disease-specific TUFM function remain to be deciphered. A central unresolved question is which specific transcription factors, epigenetic modifications, or signaling pathways exert dominant control over TUFM’s transcriptional and post-translational regulation in a given cancer context, thereby determining its functional output toward oncogenic or tumor-suppressive programs. Beyond cancer, analogous upstream networks that govern TUFM expression, stability, or localization in other pathologies—ranging from metabolic liver disease to cardiovascular and neurological disorders—also await systematic mapping.

Future research will benefit from tools such as spatiotemporal omics, high-resolution imaging, and chemical biology to resolve these questions in dynamic and systematic dimensions. Elucidating TUFM regulatory networks, integrative logic, and its context-dependent functions will not only reveal unifying principles behind its paradoxical roles across diverse diseases but also lay a solid foundation for developing precise therapeutic strategies targeting this critical node. Ultimately, deciphering TUFM’s multifaceted functions holds promise for establishing novel therapeutic paradigms to restore cellular homeostasis across a spectrum of diseases, ranging from cancer to neurodegeneration.
